# On Dental Deformities

**Published:** 1856-04

**Authors:** J. L. Levison


### ARTICLE II.

### *On Dental Deformities.* By J. L. LEVISON.*

[Continued from page 531, of Vol. 5, New Series.]

AMONG the many topics which might be discussed under the *generic* title "of dental deformities," there is one which seems not altogether unworthy the attention of your readers.

If we examine the mouth of any one who has had a partial loss of teeth, either from disease in them, or from an accident, we shall observe a certain order in the phenomena, more or less defined, in the comparative duration of the process.

If, for instance, the upper front teeth are extracted or knocked out, the corresponding incisors in the lower, will, after some length of time, present not only the whole of their crowns, but also some considerable portion of their respective fangs. And among the fallacies which have been propounded, as explanatory of the fact, it has been assumed by some superficial students, that the lower teeth, under such circumstances, continue to grow, as nature equally disliked a space, as much as she is presumed to be outraged by a vacuum! Others have made the learned attempt to show this exigency (the loss of the upper teeth) gives a new power to the lower ones, similar to the one which is present with the rodentia, namely, that they grow indefinitely for the sole purpose of rendering such an unfortunate being capable of cutting his "bread and butter," and to speak without "lisping;" and these results they ultimately accomplish! There is in the

*TO THE EDITORS.

GENTLEMEN: Having removed from Briton to 19 Dorset Place, Dorset Square, London, which has so sorely deranged my manuscripts, that they present a melancholy aspect, appearing disordered and confused, so that I am under the necessity of sending this as the concluding paper. And, probably, that some of your learned readers may be induced to take up the subject, as it is both important and at the same time of practical value, and do it greater justice than I can hope to do for some time to come. I am, gentlemen,

Yours, very truly, J. L. LEVISON.

first place, this well marked difference—that in man the crowns never grow, either as a whole, nor does the enamel seem to be renewed; for when there is any chemical action exerted on it, the substance is thinned, showing the subjacent dentine; and if a portion is broken off, the latter blackens, from being deprived of its hard and solid protection. But the rodentia, from their habit of constantly gnawing hard substances, actually *wear* away by mere mechanical friction, the sharp, chisel-like edges of their teeth, and which would have been worthless as instruments, if there had not been a provision made for the renewal of the enamel cutting edge, and we find that such is the case during their growth and maturity. That perspective wisdom is manifested in this as in all the works of the Creator. We may further remark, that by the mechanism of the jaws of the rodentia, the front teeth form a most complete cutting apparatus. For behind the central incisors, there are placed two short teeth, with a space, so that when the upper ones shut, they fit in a well fitted groove, their sharp cutting edges chiseling the pieces without any great effort.*

Now in the particular case we propose examining, there is not any actual increase in either the crowns or the fangs, but the elongation is a provision to render the injury to the health of the individual less than it might otherwise be, if there remained an incapacity for teeth to be antagonized, and which depend solely on their actual approximation; otherwise they lose all their vitality in the buccal cavity.

The proximate cause for the elongation seems to be, the absence of resistance in the antagonizing instruments, and the elongation of the incisors is a provision in order to render the individual less inconvenienced.

For, we observe that when the resisting power is no longer exerted (as in the case of the loss of the upper incisors, or when any of the molars are removed) the consequences are the simple result of there being no longer any antagonism. It is then that the remaining teeth, the consequences of those which have been

* There is not a doubt, but this beautiful contrivance suggested the method by which a blacksmith separates a piece of heated iron on a chisel-like wrench.

removed, though they do not grow, there is in effect a process which acts as an equivalent. The alveolar processes, of the teeth which are deprived of the resisting power of those with which they have acted in the normal condition of jaws, gradually contract from below, and hence the fangs are raised or pushed forwards by degrees, until the crowns find a resistance against the absorbed alveoli covered with the gums, in either jaws in which there is an absence of the corresponding teeth; and thus there is some compensation for the loss of the teeth either destroyed by disease or accident.

The final cause of this arrangement is rendered obvious by the fact that when the remaining teeth, ultimately, come in contact with the sockets, which have become consolidated, they are assisted in some slight degree in the manducatory process. And hence, when nature is the dentist, there is a compensation for the previous injury. And we are led to infer that she never contemplated artificial teeth as a better substitute! But as man is endowed with intellectual powers fitting him to be, not only a student but also as an interpreter of the laws of his wonderful organism and of all things in the outer world, that it may be presumed he was intended to apply his knowledge, however derived, for his own advantage.

It is, therefore, not surprising that he should have attempted to compensate for accidents or injuries from disease, when either affected the symmetry or the usefulness of any part of his body. Hence, the rude attempt in the early periods of society to supply the loss of teeth; particularly by means of rudely-formed blocks of hard wood, such as the *lignum-vitæ*, nicely cut and adjusted to fill up the spaces between the remaining molars.* The wisdom of this plan may be inferred by the fact, that when one or more of the molar teeth are extracted, the separated teeth cannot approximate, because there exists an insuperable obstacle, from the irregular absorption of the alveolar processes in which the lost grinders had had

"A local habitation."

* This was actually the case in one of the mummies taken from a tomb in Thebes.

Sometimes, for example, the absorption may take place, giving a tendency to the anterior edge of the socket to approximate to the posterior edge, or *vice versa.* In either case there exists a bony-ridge as a barrier. If on the other hand, the absorption takes place from each side of the empty alveolus, the ridge is curved in a line with the remaining teeth. In which case their crowns may approximate, whilst the space between the necks of such teeth is nearly the same as when all the molars had been in situ. The consequence of this divergence is the cause of more or less havoc than merely the loss of one or two grinders. As the parts of the teeth which should cross each other (the incisors and cuspidati) and the molars which should meet in antagonism, are thrown, by this one circumstance, out of their natural symmetry, this interferes with the proper working of the whole dental apparatus.

It is then on this very account that artificial teeth are recommended as a *dernier resort*, to keep the jaws in such a condition that their symmetry and mechanical motions may neither be impeded nor distorted. And this rule particularly applies to the restoration of any of the molars. For by a natural action of the jaws, they are drawn forcibly together by the temporal muscles, when the molars in both the upper and under have their broad surfaces brought in contact with great force, and by a marvellous mechanical contrivance, move with a springy motion in their respective sockets. This simple and beautiful arrangement to obviate the effect of concussion, has suggested the buffers of the railway carriage.

In the application of the compensating principle, as applied to the teeth, there is effected all the advantage of actual contact, without the risk of breaking the hard enamel of them, which must have resulted from the natural brittleness of this crystalline substance, had such provision for their safety been withheld. Besides, which, if the force of the collision had not been broken on the motion to which allusion has been made, the vessels of the periosteum would have been liable to serious injury. There is another advantage from this most excellent and most perfect arrangement. The lower jaw having a free motion has been compared, by Richerand and others, to a hammer; and the immovable upper jaw, to an anvil. Whilst the actual force in the jaws of a strong man, taking the various muscles into consideration, essential to produce their compound action, it is said to be equal to four hundred weight crushing power!

If, therefore, there is a loss of the molars, from the jaws being then incapable of that antagonizing action we have spoken of—the lower jaw must strike with an extraordinary force the incisors, and so injure them by this unnatural shaking, that their continued existence is greatly jeopardised. From these general observations it will be obvious, that I advocate the aid of art in cases of loss of one or more of the molars, but not affixed to plates, either of gold, platina or palladium, and this for two reasons.

1st. Because they must be fixed by clasps of the same metal.

2d. That any metal, from being alloyed, must be liable to be oxydised, which increases the tendency to produce some galvanic action, more or less affecting the chemical quality of the saliva, by converting its free oxygen into an acid, *sui generis*, the effect of which is gradually rendered manifest by the removal of the earthy-portion of the remaining teeth.

These consequences are obviated by using blocks of the tusk of the hippopotamus. And even if their grinding surfaces wear away rapidly, or if they become blackened—there is avoided all galvanic action, and there is preserved intact the perfect flavor of the food, which is not the case when any metal is used.

I, therefore, enter my deep protest against all who boast that artificial teeth are equal to those of nature. They cannot be. It is a physical and mechanical impossibility. For the natural teeth being fixed in a receptacle, and by a most simple and marvellous contrivance are thus enabled to tear, cut and crush the aliment, without injury to themselves or their "local organs;" it is impossible to supply any artificial substitute to ensure these advantages and immunities. For all artificial teeth must be fixed so as to press on the living gums, which are liable to be affected with more or less inflammatory action, as a result of a foreign agent acting mechanically on them. And in the case where there seems no immediate evidence that such is the case, time demonstrates the contrary. That there has been a *chronic* and not an *acute* affection by the gradual absorption of the substance of the gums and the subjacent bone.

The hippopotamus blocks are less injurious in effecting these results, than when any metallic base is used. Hence, even the former, although but imperfect substitutes, are still useful in preventing more complex injury and certain deformity, and may be regarded among the useful arts.

But when advertising *charlatans* compare them with nature's handiwork, nay, who even affirm that they are superior, then, indeed, we cannot help regarding all such artificial productions as mere miserable substitutes, suggested by a strong necessity, but still defective when compared with those which formed a part and parcel of the living organism, and which were intended by the divine and infinite Creator to be *permanent;* and as they are not so, is but one among many other penalties we pay for our state of civilization.

19 *Dorset Place, Dorset Square, London, Jan.* 10*th,* 1856.

